# Clinical Characteristics, Laboratory Findings, and Prognosis in Patients With *Talaromyces marneffei* Infection Across Various Immune Statuses

**DOI:** 10.3389/fmed.2022.841674

**Published:** 2022-04-15

**Authors:** Dianwu Li, Huaying Liang, Yiqun Zhu, Qinyu Chang, Pinhua Pan, Yan Zhang

**Affiliations:** ^1^Department of Respiratory Medicine, National Key Clinical Specialty, Branch of National Clinical Research Center for Respiratory Disease, Xiangya Hospital, Central South University, Changsha, China; ^2^Center of Respiratory Medicine, Xiangya Hospital, Central South University, Changsha, China; ^3^Clinical Research Center for Respiratory Diseases in Hunan Province, Changsha, China; ^4^Hunan Engineering Research Center for Intelligent Diagnosis and Treatment of Respiratory Disease, Changsha, China; ^5^National Clinical Research Center for Geriatric Disorders, Xiangya Hospital, Changsha, China

**Keywords:** *Talaromyces marneffei*, clinical characteristics, prognosis, next-generation sequencing, immune status

## Abstract

**Objective:**

*Talaromyces marneffei* (TM) is an opportunistic fungus that is predominantly prevalent among patients who are HIV-positive in South-East Asia. However, few studies focused on the clinical features, laboratory findings, and prognosis across varying immune states.

**Methods:**

A total of 54 patients with TM infection in Xiangya Hospital of Central South University from January 1, 2006 to October 31, 2021 were retrospectively analyzed. Clinical profiles were compared across the different immune statuses by HIV-positive (HIV group, *n* = 18), HIV negative but with immunocompromised conditions (Non-HIV with IC Group, *n* = 11), and immunocompetent patients (*n* = 25).

**Results:**

All the patients were diagnosed by pathogen culture or by metagenomic next-generation sequencing (mNGS). The median age was 50, and patients with HIV were much younger compared to the other two groups. The most common symptom at presentation was fever (79.6%), followed by cough (70.4%), weight loss (61.1%), and expectoration (53.7%). The patients with HIV were more likely to develop into a subtype of disseminated TM affecting multiple organs including lymph node, liver, skin, and spleen, thus, resulting in higher hospital mortality compared to the other two groups. Patients without HIV but with immunocompromised conditions presented similar hospital mortality rates compared to immunocompetent patients, while experiencing longer days of hospitalization to recover from the diseases. Additionally, in this study, the pathogen culture easily confirmed the patients with HIV. However, mNGS presented as a promising tool to confirm TM infection in those suspicious patients without HIV.

**Conclusions:**

In summary, patients with HIV were more likely to develop into disseminated TM, resulting in higher mortality compared to those patients without HIV. Additionally, mNGS presented as an important supplementary tool to confirm TM infection in patients without HIV, particularly in those with immunocompromised diseases.

## Introduction

*Talaromyces marneffei* (TM) is an opportunistic and highly invasive fungus endemic, that is predominantly restricted to Southeast and Eastern Asia (1, 2). It was originally isolated from a bamboo rat in Vietnam in 1956 (3) and was firstly reported with natural human infection in 1973 (4). It is one of the most important thermally dimorphic pathogenic fungi (5) and grows as a mycelium at 25°C and as yeast at 37°C due to the organism's principal virulence factors (6). A hypothetical diagnosis of TM infection is made on clinical findings in conjunction with microscopic identification of characteristic intracellular TM yeast cells, while definitive diagnosis depends on the isolation of TM by culture from suspicious clinical specimens (7). The TM presents as a unicellular organism with round to oval cells under microscopic observation (8). In recent years, mNGS has been a promising tool to confirm TM infection for suspicious individuals as well.

The TM infection commonly occurs in patients with human immunodeficiency virus infection (HIV), but in recent years, more and more TM infection has been diagnosed in patients without HIV, and even in immunocompetent individuals (9, 10). However, studies on clinical characteristics, laboratory findings, and prognosis in patients with TM infection with varying immune statuses are still limited. In the current study, we conducted a retrospective analysis on demographics, clinical symptoms, laboratory and imaging presentation, and prognosis related to TM infection in patients with varying immune statuses.

## Methods

### Patients

We retrospectively extracted and analyzed the clinical profile data on patients with TM infections in Xiangya Hospital of Central South University from January 2006 to October 2021. This study was approved by the Institutional Review Boards (IRBs) in Xiangya Hospital, Central South University (No. 202104005). Clinical profiles including demographics, comorbidities, clinical symptoms, laboratory and imaging findings, and prognosis were obtained from the electronic record. The TM infection was diagnosed by experienced doctors, combining suspicious clinical manifestations with one of the following evidence (11–13):

a. Pathogen culture: Using standard culture techniques to isolate TM from clinical specimens. The crucial evidence of the positive TM was its thermal dimorphism, that is, the form of mycelium at 25°C coupling with brick red pigment and the form of yeast at 37°C.
b. After Wright staining, Giemsa staining, or periodic acid Schiff staining of cytology and histopathology specimens, the special yeast form and diaphragm of TM are directly observed with the microscope.
c. Genetic methods: Genetic methods including mNGS (in the current study) and other genetic tests illustrate that TM is positive.

### Definitions

Imaging and physical exam findings, positive culture site, and histopathology were commonly used to define sites of infection. If patients had only one involved lesion or organs without systemic symptoms or physical signs, that would be defined as focalized TM infection. When the existence of TM is confirmed in blood or bone marrow, or more than one viscus with systemic symptoms, that would be defined as disseminated TM infection. Immune status was divided into three groups as following criteria: (1) HIV group was defined as patients accompanied with HIV infection; (2) Non-HIV with IC group was defined as those patients without HIV, but with immunocompromised conditions as any of the following situation: progressed malignancy, chemotherapy, solid organ or a stem-cell transplant, or immunosuppressive medications including corticosteroids, biologic immunosuppressants, etc.; (3) the immunocompetent group was classified as those patients without HIV or absence of an immunocompromised condition.

### Statistical Analysis

All data in the current study were analyzed with SPSS 26.0 software (IBM SPSS Inc., USA). Continuous variables with skewed distribution were presented as medians and interquartile ranges and compared by the Kruskal-Wallis H test. Categorical variables were expressed as frequencies and percentages (%) and analyzed by the Chi-square test or Fisher's exact test. A value of *P* < 0.05 was considered statistically significant.

## Results

### Clinical Characteristics of TM Infection in Patients With Various Immune Statuses

This retrospective analysis included 54 patients with TM-infection in Xiangya Hospital of Central South University from January 1, 2006 to October 31, 2021. All the patients in the study were identified by pathogen culture or mNGS. The gender composition was different between the three groups, with males accounting for 88.9, 54.5, and 88.0% in the HIV, Non-HIV with IC, and Immunocompetent groups, respectively. The median age was 50, and patients with HIV were much younger compared to the other two groups (*P* = 0.02). The most common symptom at presentation was fever (79.6%), followed by cough (70.4%), lymphadenopathy (70.4%), weight loss (61.1%), expectoration (53.7%), skin lesion (50%), gastrointestinal symptoms (33.3%), splenomegaly (31.5%), hepatomegaly (35.2%), bone destruction (11, 20.4%). The patients with HIV were more likely to develop into a subtype of disseminated TM, affecting multiple organs including lymph node, liver, skin and spleen, and so on (Table 1).

**Table 1:** Clinical characteristics of the patients with TM infection across the three groups.

	**HIV** ***N =* 18**	**Non-HIV with IC** ***N =* 11**	**Immunocompetent** ***N =* 25**	**Total** **(*N =* 54)**	***P***
**Median age, y**,	38.0(28.0–50.5)	52.0(32.0–57.0)	55.0(44.5–62.5)	50.0(36.0–58.0)	0.020
**Male**	16(88.9%)	6(54.5%)	22(88.0%)	44(81.5%)	0.049
Male	16(88.9%)	6(54.5%)	22(88.0%)	44(81.5%)	
Female	2(11.1%)	5(45.5%)	3(12.0%)	10(18.5%)	
Smoking	11(61.1%)	4(36.4%)	13(52.0%)	28(51.9%)	0.433
Comorbidity	16(88.9%)	11(100.0%)	20(80.0%)	47(87.0%)	0.332
Complication	17(94.4%)	5(45.5%)	19(76.0%)	41(75.9%)	0.012
**Season characteristic**					
Spring	3(16.7%)	4(36.4%)	4(16.0%)	11(20.4%)	0.371
Summer	6(33.3%)	5(45.5%)	9(36.0%)	20(37.0%)	0.801
Autumn	4(22.2%)	1(9.1%)	8(32.0%)	13(24.1%)	0.385
winter	5(27.8%)	1(9.1%)	4(16.0%)	10(18.5%)	0.481
**Stage of disease**					0.003
Disseminated Disease	18(100.0%)	6(54.5%)	16(64.0%)	40(74.1%)	
Localized Disease	0(0.0%)	5(45.5%)	9(36.0%)	14(25.9%)	
**Immunosuppression**
Active Malignancy (solid organ)	0(0.0%)	1(9.1%)	0(0.0%)	1(1.9%)	0.204
Active Malignancy (hematologic)	0(0.0%)	1(9.1%)	0(0.0%)	1(1.9%)	0.204
Chemotherapy	0(0.0%)	1(9.1%)	0(0.0%)	1(1.9%)	0.204
Corticosteroids	0(0.0%)	9(81.8%)	0(0.0%)	9(16.7%)	<0.001
Other immunosuppressor	0(0.0%)	4(36.4%)	0(0.0%)	4(7.4%)	0.001
**Symptoms and signs**
Fever	17(94.4%)	8(72.7%)	18(72.0%)	43(79.6%)	0.148
Cough	14(77.8%)	8(72.7%)	16(64.0%)	38(70.4%)	0.638
Expectoration	9(50.0%)	6(54.5%)	14(56.0%)	29(53.7%)	0.925
Hemoptysis	1(5.6%)	1(9.1%)	2(8.0%)	4(7.4%)	1.000
Dyspnea	0(0.0%)	0(0.0%)	2(8.0%)	2(3.7%)	0.412
Chest pain	2(11.1%)	1(9.1%)	6(24.0%)	9(16.7%)	0.448
Bone destruction	3(16.7%)	1(9.1%)	7(28.0%)	11(20.4%)	0.459
Arthralgia	1(5.6%)	2(18.2%)	2(8.0%)	5(9.3%)	0.585
Skin lesion	14(77.8%)	5(45.5%)	8(32.0%)	27(50.0%)	0.012
Weight loss	10(55.6%)	5(45.5%)	18(72.0%)	33(61.1%)	0.293
Gastrointestinal symptoms	9(50.0%)	4(36.4%)	5(20.0%)	18(33.3%)	0.111
Lymphadenopathy	17(94.4%)	6(54.5%)	15(60.0%)	38(70.4%)	0.015
Hepatomegaly	10(55.6%)	1(9.1%)	6(24.0%)	17(31.5%)	0.022
Splenomegaly	10(55.6%)	2(18.2%)	7(28.0%)	19(35.2%)	0.090

*IC, Immunocompromise*.

### Laboratory and Image Findings in TM Infection Across Various Immune Statuses

**Figure 1:**
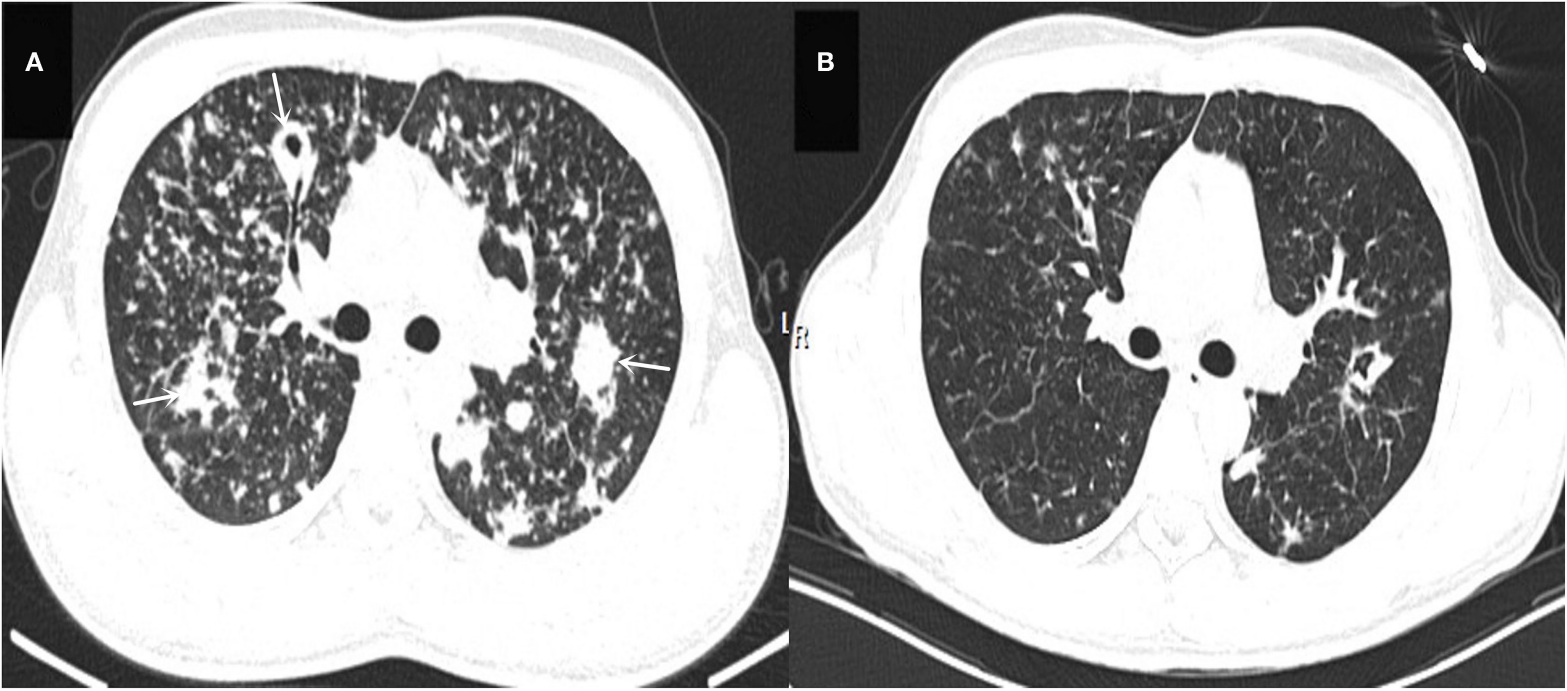
**(A)** Shows the patient's lung CT presentation on admission (white arrows). **(B)** Shows a CT of the lungs reviewed after 2 months of anti-TM treatment, which is significantly better than **(A)** in terms of absorption.

**Figure 2:**
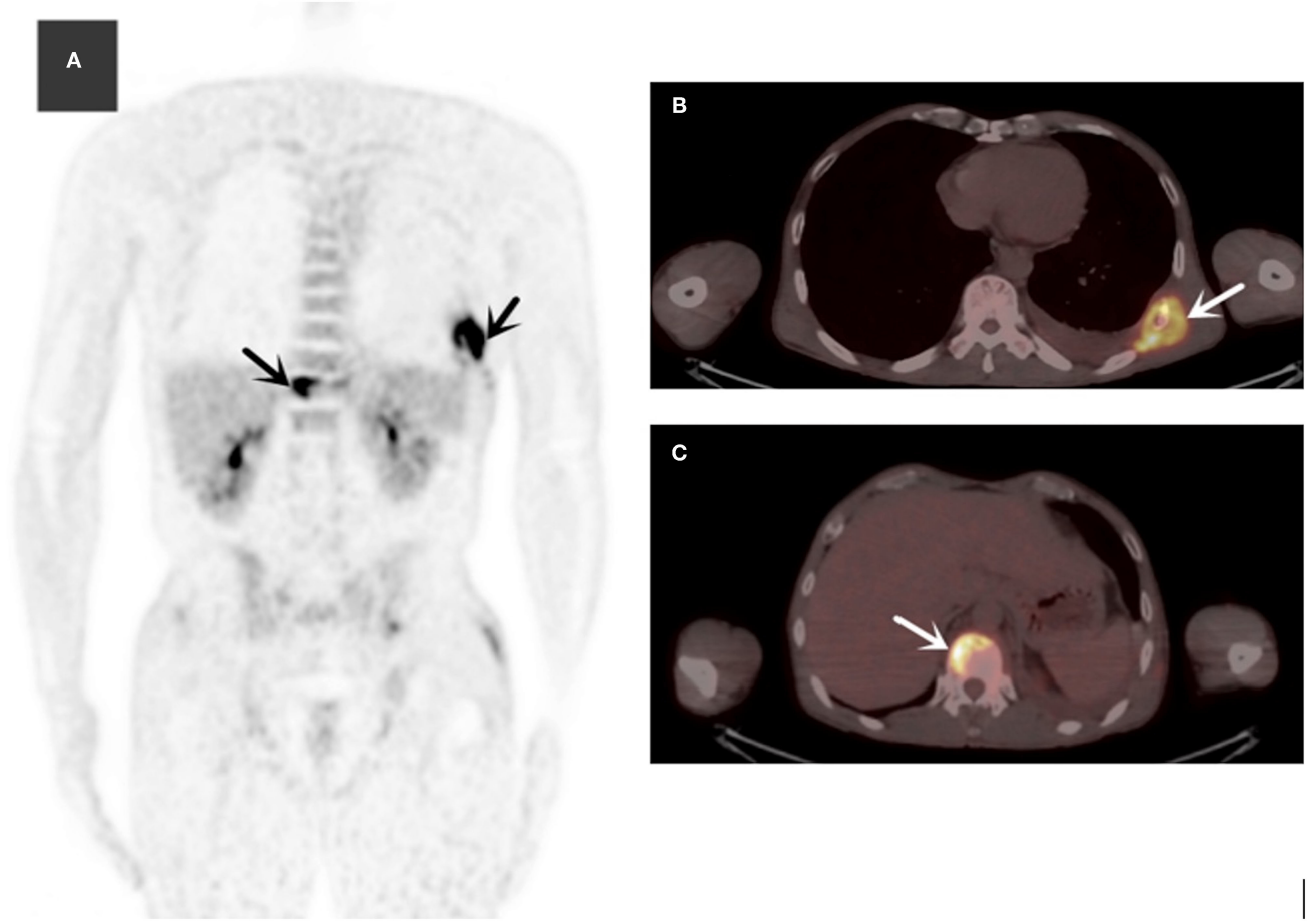
The MIP (maximum intensity projection) of 18F-FDG (fluoro-D-glucose) PET/CT **(A)** revealed multiple hypermetabolic lesions in the whole body (black arrows). Axial slices showed normal ribs **(B)** (SUVmax = 10.3) and multiple hypermetabolic lesions of the spine **(C)** (white arrows).

The laboratory and image findings were presented in Table 2. Compared to immunocompetent patients, patients with or without HIV with other immunocompromised conditions had a lower number of leukocytes, platelets (PLT), lymphocytes, and eosinophils that reflected their immune status. Compared to patients in the immunocompetent group, patients with HIV had significantly lower albumin and uric acid (UA), but with higher alanine transaminase (ALT), aspartate transaminase (AST), lactate dehydrogenase (LDH), total bilirubin (TBil), and direct bilirubin (DBil). Patients with HIV, or otherwise immunocompromised conditions, showed obvious immune dysfunction based on the impairment of CD4, CD8, and complement levels of C3 and C4. From the image findings, almost all patients with TM infections had lesions in their lungs, indicating respiratory airway was the main entry route for TM infection. Several cases were also accompanied by a skin infection, and one of them got the infection after skin surgery, then spread to the lungs, and represented with bilateral diffuse nodular lesions (Figure 1A), which were significantly absorbed after anti-TM treatment with Amphotericin B (Figure 1B). Additionally, there is also a tendency for multiple invasions throughout the body in patients without HIV, which can easily be misdiagnosed as advanced malignancies. In the study, one patient was admitted with generalized pain and a subcutaneous mass on the left posterior back, PET-CT (Figure 2) gave a hint of multiple possible malignant lesions, but TM was confirmed by pathogen culture for the tissue biopsy.

**Table 2:** Laboratory findings and imaging in patients with TM infection among various immune statuses.

	**HIV** ***N =* 18**	**Non-HIV with IC** ***N =* 11**	**Immunocompetent** ***N =* 25**	**Total** ***N =* 54**	***P***
**Laboratory findings**
WBC ( ×10^∧^9/L)	5.3(3.6–6.7)	5.9(2.9–8.7)	9.9(4.6–14.1)	6.4(4.4–11.6)	0.016
Hb(g/L)	95.5(82.0–103.5)	88.0(75.0–107.0)	103.0(84.0–119.5)	97.0(80.7–111.0)	0.362
PLT ( ×10^∧^9/L)	116.0(44.5–176.0)	150.0(22.0–312.0)	231.0(142.5–287.0)	158.0(53.5–265.5)	0.031
Lymphocytes ( ×10^∧^9/L)	0.3(0.1–0.4)	0.6(0.3–1.1)	1.4(0.9–2.0)	0.7(0.3–1.4)	<0.001
Lymphocytes (%)	5.0(4.0–8.2)	9.3(8.0–21.6)	14.3(10.1–21.2)	10.9(5.7–17.7)	<0.001
Neutrophils ( ×10^∧^9/L)	4.7(3.2–6.2)	5.2(2.1–8.1)	5.6(3.1–11.4)	5.0(3.0–8.5)	0.272
Neutrophils (%)	90.5(81.9–93.4)	79.7(74.6–87.9)	73.8(66.7–81.9)	79.7(7.3–88.5)	<0.001
Eosnophils ( ×10^∧^9/L)	0.0(0.0–0.1)	0.0(0.0–0.1)	0.2(0.1–0.4)	0.1(0.0–0.2)	0.001
Eosnophils (%)	0.4(0.0–1.0)	0.4(0.0–2.1)	2.1(0.7–3.7)	1.0(0.1–2.5)	0.004
Albumin (g/L)	24.2(19.7–29.1)	28.4(25.3–34.7)	31.1(27.9–35.8)	28.7(24.6–33.9)	0.002
Globulin (g/L)	35.7(27.9–43.1)	26.0(23.9–33.5)	43.7(33.3–46.7)	35.8(27.4–44.7)	0.001
ALT(U/L)	40.5(33.0–75.0)	53.4(11.9–73.3)	18.9(11.4–43.4)	34.3(14.9–66.0)	0.036
AST(U/L)	136.0(65.4–215.0)	45.0(14.5–61.0)	21.5(16.1–33.3)	45.0(16.8–123.5)	<0.001
BUN(mmol/L)	4.1(3.8–6.8)	5.3(3.8–7.3)	4.4(3.2–7.4)	4.4(3.5–7.1)	0.873
SCr (umol/L)	75.0(65.0–91.1)	74.0(51.2–99.0)	74.0(63.2–96.5)	74.5(62.6–94.9)	0.827
UA (umol/L)	216.0(154.5–342.5)	359.0(199.9–469.0)	318.0(243.0–385.5)	301.0(200.4–380.5)	0.045
TBil	14.9(10.4–29.7)	6.0(5.4–8.3)	10.0(5.05–15.25)	10.1(5.6–15.4)	0.008
DBil	5.9(3.2–16.1)	2.5(2.1–4.3)	4.2(2.8–6.9)	4.3(2.3–6.9)	0.028
LDH(U/L)	574.0(317.7–800.5)	317.0(190.0–449.0)	178.5(147.2–260.2)	267.0(177.0–507.0)	<0.001
CK(U/L)	37.5(29.5–91.9)	20.8(18.8–81.0)	32.2(22.9–58.6)	32.9(22.7–62.6)	0.204
CK–MB(U/L)	13.3(10.4–20.2)	11.2(10.1–17.3)	10.7(7.9–16.8)	11.4(9.5–17.0)	0.434
Mb(U/L)	24.9(18.7–43.9)	19.0(14.5–45.0)	22.7(15.6–35.7)	23.8(16.0–39.2)	0.665
PT(S)	13.1(12.6–14.9)	12.6(11.4–16.8)	14.2(12.4–15.8)	13.3(12.4–15.2)	0.416
APTT(S)	43.8(38.6–48.5)	32.8(25.4–40.4)	38.8(33.5–45.3)	39.0(33.0–45.4)	0.053
TT(S)	20.7(18.8–22.8)	19.0(17.9–20.3)	18.0(16.9–18.8)	18.7(17.8–20.9)	0.002
FIB(g/L)	2.7(1.6–3.9)	3.7(2.8–4.9)	3.5(2.5–6.2)	3.6(2.2–4.5)	0.200
D – dimer	1.7(1.2–3.1)	0.3(0.3–1.1)	0.5(0.2–1.2)	0.8(0.3–2.0)	0.004
CD4+T cell(a/ul)	4.8(3.0–15.0)	109.0(109.0–109.0)	343.0(157.0–469.0)	107.0(7.4–358.0)	0.074
CD4+T cell(%)	3.8(0.7–14.0)	27.4(22.7–68.7)	38.1(27.7–45.8)	33.5(11.4–42.6)	0.062
CD8+T cell(a/ul)	225.0(205.0–284.0)	66.1(16.3–66.1)	159.0(110.0–596.0)	182.0(111.5–269.2)	0.200
CD8+T cell(%)	71.0(51.3–75.0)	49.0(29.0–49.0)	26.4(19.4–46.5)	37.6(23.0–69.0)	0.098
CD4+ / CD8+T cell	0.0(0.0–0.1)	0.9(0.3–4.3)	1.5(0.7–2.4)	1.0(0.1–2.1)	0.032
C3 (mg/L)	651.0(265.0–953.0)	658.5(353.2–775.2)	961.0(756.0–1247.5)	747.0(513.0–1070.0)	0.008
C4 (mg/L)	200.0(51.6–272.0)	173.5(98.2–243.2)	248.5(211.5–313.0)	225.0(136.0–272.0)	0.044
IgG (g/L)	14.9(12.6–23.0)	10.6(7.5–14.1)	21.0(15.6–28.2)	15.6(12.4–23.2)	0.004
IgA (g/L)	4,700.0(2,550.0–6,760.0)	1,504.5(637.7–2,260.0)	2,315.0(1,302.5–3,457.5)	2,460.0(1,340.0–4,230.0)	0.001
IgM (g/L)	825.5(569.5–1,402.5)	1,130.0(691.0–1,657.5)	946.5(765.0–1,457.5)	946.5(689.2–1,402.5)	0.713
CRP (mg/L)	68.5(43.0–103.2)	34.5(14.8–73.4)	40.7(20.0–119.5)	53.0(24.0–101.0)	0.156
ESR (mm/h)	53.0(28.5–83.0)	33.0(26.5–78.5)	61.0(39.5–103.0)	53.0(33.0–96.0)	0.340
PCT (ng/ml)	2.3(0.6–6.6)	0.2(0.0–0.9)	0.2(0.0–0.8)	0.5(0.1–2.9)	0.008
**Imaging**
Unilateral Infiltrate or Consolidation	5(29.4%)	0(0.0%)	2(8.3%)	7(14.0%)	0.107
Bilateral Infiltrate or Consolidation	12(70.6%)	9(100.0%)	22(91.7%)	43(86.0%)	0.107
Pulmonary Nodule	8(47.1%)	3(27.3%)	9(36.0%)	20(37.7%)	0.533
Cavitary Lesion	0(0.0%)	2(18.2%)	5(20.8%)	7(13.7%)	0.148
Hilar Lymphadenopathy	5(29.4%)	0(0.0%)	3(12.5%)	8(15.4%)	0.137
Mediastinal Lymphadenopathy	11(64.7%)	0(0.0%)	9(37.5%)	20(38.5%)	0.001
Intra-Abdominal Lymphadenopathy	14(100.0%)	1(11.1%)	3(13.6%)	18(40.0%)	<0.001
Splenic Infiltrate	10(55.6%)	1(9.1%)	6(24.0%)	17(31.5%)	0.022
Liver Infiltrate	10(55.6%)	2(18.2%)	7(28.0%)	19(35.2%)	0.090
CNS Findings	0(0.0%)	0(0.0%)	1(4.0%)	1(1.9%)	1.000

### Treatment Strategies and Prognosis of TM Infection Across Various Immune Statuses

The treatment strategies and prognosis in the patients with TM infection was presented in Table 3. Patients with TM with HIV had the highest hospital mortality rate among the three groups. Those patients without HIV with immunocompromised conditions had a similar hospital mortality rate with immunocompetent patients, but experienced longer days of hospitalization. Additionally, patients with HIV were confirmed easier by the pathogen culture, while a proportion of patients without HIV were diagnosed by mNGS. In the study, 15 suspicious patients were examined with pathogen culture and mNGS. The results showed 5 patients with double-positive results by culture and mNGS, while 9 patients showed positive mNGS but negative culturing results (Supplementary Figure 1), only 1 patient had positive culturing result but negative mNGS (Table 4). This interesting phenomenon indicated that, to some extent, mNGS had much higher sensitivity compared to conventional pathogen culture for TM infection, particularly in those patients without HIV.

**Table 3:** Treatment strategies and prognosis in patients with TM infection.

	**HIV** ***N =* 18**	**Non-HIV with IC** ***N =* 11**	**Immunocompetent** ***N =* 25**	**Total** ***N =* 54**	***P***
Multiple infections	4(22.2%)	8(72.7%)	8(32.0%)	20(37.0)	0.022
**Prognosis**
Hospital stays(d)	9.5(5.0-13.5)	33.0(13.0-38.0)	18.0(12.5-31.5)	15.0(10.0-26.5)	<0.001
Hospital mortality	8(44.4%)	2(18.2%)	2(8%)	18(22.2%)	0.012
**Diagnosis**
mNGS	0(0.0%)	7(63.6%)	7(28.0%)	14(25.9%)	<0.001
Positive culture results	18(100.0%)	7(63.6%)	20(80.0%)	45(83.3%)	0.015
**Positive site (% of those with positive culture and mNGS)**
blood	13(72.2%)	4(36.4%)	5(20.0%)	22(40.7%)	0.003
Purulent discharge of the skin	0(0.0%)	2(18.2%)	8(32.0%)	10(18.5%)	
Respiratory tract	3(16.7%)	5(45.5%)	10(40.0%)	18(33.3%)	
Bone Marrow	2(11.1%)	0(0.0%)	0(0.0%)	2(3.7%)	
Cerebrospinal Fluid	0(0.0%)	0(0.0%)	1(4.0%)	1(1.9%)	

**Table 4:** Comparison of next-generation sequencing (NGS) and traditional culture analyses in TM infection.

**NGS**	**Culture**		**Total**
	**+**	**–**	
**+**	5	9	14
**–**	1	0	1
**Total**	6	9	15

## Discussion

In the study, we compared the clinical characteristics, laboratory findings, and prognosis of TM-infected based on immune status and found patients with HIV were more likely to develop into disseminated TM and had the highest mortality rate. Those patients without HIV but with immunocompromised conditions had a similar hospital mortality rate with immunocompetent patients, while experiencing longer days of hospitalization. Additionally, mNGS shows promising potential as a tool to confirm TM infection, particularly in those patients without HIV, or with immunocompromised conditions.

In China, TM is predominantly popular in several southern provinces, such as Guangxi, Guangdong, and Yunnan Province (14), and the number of TM cases has been dramatically increasing in mainland China in the last decades (15). The TM infection has been commonly found in patients with immunocompromised and impaired cell-mediated immunity, including secondary immunodeficiency due to HIV infection, progressed malignancy, and immunosuppressive therapy (16, 17). However, recently, an increasing number of cases of TM infection have been reported in individuals without HIV, even without obvious immunocompromised conditions (18–20). In the current study, only one-third of patients with TM were accompanied by HIV infection, which was less frequent than the previously studied (21), and almost one-half of patients with TM infection were immunocompetent patients without HIV or obvious immunosuppression. This observation may be explained by the following reasons: (1) with the use of potent antiretroviral therapy, the incidence of HIV-associated TM infection decreased correspondingly; (2) new therapies for autoimmune diseases, and an increasing prevalence of solid organ and bone marrow transplants have shifted the epidemiology of certain fungal infections; (3) the developing diagnostic technology with gene sequence might reverse the previous condition that patients without HIV infection were underdiagnosed. It also demonstrates that more attention should be paid to those patients with suspicious TM infection even though they are not accompanied with HIV or otherwise immunocompromised conditions.

Our study showed that more patients were infected during the period from April to September of the year, which is the rainy season in Hunan province. Meanwhile, a seasonal pattern with increasing incidence of TM infection during the rainy seasons has been observed in northern Thailand before (22, 23). It might be that the heavy rainfall provides a favorable condition for the growth of the fungus, thus, increasing the chance of exposure to susceptible hosts (23). Most patients with TM presented with similar symptoms with previous reports (24), including fever, weight loss, cough, lymphadenopathy, cutaneous lesions, and hepatosplenomegaly. The respiratory system was commonly involved in TM infection, with productive cough, dyspnea, and hemoptysis as common symptoms. Therefore, TM infection should be differentiated from other fungal lung infections for effective antifungal therapies, such as pulmonary candidiasis, Pneumocystis pneumonia, and so on (25, 26). Patients with HIV were more likely to develop into disseminated TM and had the highest mortality rate. Those patients that are negative of HIV with immunocompromised conditions had a similar hospital mortality rate with immunocompetent patients, while experiencing longer days of hospitalization.

An interesting finding was that all patients with HIV in the study were diagnosed by positive TM culture, while 63.6% of patients without HIV with immunocompromised conditions and 28% of immunocompetent patients were diagnosed by mNGS with negative culture results. This phenomenon could be explained by the following reasons: (1) Patients with HIV had worst immunity and more possibility to disseminate into blood compared to the other two groups, thus, greatly increasing the chance of obtaining a positive culture result from the blood samples; (2) the sensitivity of positive TM by pathogen culture could be affected by the complicated history of medication and multiple pathogen infections in those patients without HIV but with immunocompromised diseases. To further confirm the 9 patients who were diagnosed with mNGS but without positive colonies, their clinical characteristics, treatment procedure, and prognosis coincided with TM infection (Supplementary Figure 1). Thus, it reflected that mNGS had much higher sensitivity compared to conventional pathogen culture, and it could be an important tool to detect TM infection to reduce the misdiagnosis, delay in diagnosis, and mortality, particularly in those patients without HIV but with immunocompromised conditions.

Our research had a few limitations. Firstly, this study was a single-center and retrospective study, so we involved a very small part of patients with TM infection in Hunan. There was a relatively small proportion of immunocompromised patients without HIV, thus, introducing outcome bias. A large cohort study is needed to further affirm our conclusions. Secondly, the accurate identification of TM should combine colonies (culture positive) and PCR sequencing, however, the PCR test was not applied as a conventional test in most hospitals including ours.

## Conclusion

In summary, patients with HIV were more likely to develop into disseminated TM and resulting in higher mortality compared to those patients without HIV. Additionally, mNGS presented as an important supplementary tool to confirm TM infection in patients without HIV, particularly in those patients with immunocompromised diseases.

## Data Availability Statement

The original contributions presented in the study are included in the article/Supplementary Material, further inquiries can be directed to the corresponding authors.

## Ethics Statement

The studies involving human participants were reviewed and approved by the Institutional Review Boards (IRBs) in Xiangya Hospital, Central South University. The patients/participants provided their written informed consent to participate in this study. Written informed consent was obtained from the individual(s) for the publication of any potentially identifiable images or data included in this article.

## Author Contributions

DL and HL were involved in the design of the study, collection of research data, data collation and analysis, and writing of the manuscript. YZha and PP were involved in the design of the study and review of the article, funding support and other aspects of supervision. YZhu and QC were involved in the collection of research data and writing of the manuscript. All authors contributed to the article and approved the submitted version.

## Funding

This work was supported by the Natural Science Foundation of China (Nos. 82100037 and 81770080), National Key R&D Program of China (No. 2016YFC1304204), National Science Foundation for Post-doctoral Scientists of China (No. 2021TQ0375), Hunan Outstanding Postdoctoral Innovative Talents Program (No. 2021RC2018), Key R&D Program of Hunan Province (No. 2022SK2038), and Youth Foundation of Xiangya Hospital (No. 2020Q06).

## Conflict of Interest

The authors declare that the research was conducted in the absence of any commercial or financial relationships that could be construed as a potential conflict of interest.

## Publisher's Note

All claims expressed in this article are solely those of the authors and do not necessarily represent those of their affiliated organizations, or those of the publisher, the editors and the reviewers. Any product that may be evaluated in this article, or claim that may be made by its manufacturer, is not guaranteed or endorsed by the publisher.
