# Supplementary figures and images for: Clinical Characteristics, Laboratory Findings, and Prognosis in Patients With Talaromyces marneffei Infection Across Various Immune Statuses
Source: Front Med (Lausanne). 2022 Apr 15;9:841674. doi: 10.3389/fmed.2022.841674 (PMC9051250; doi:10.3389/fmed.2022.841674)

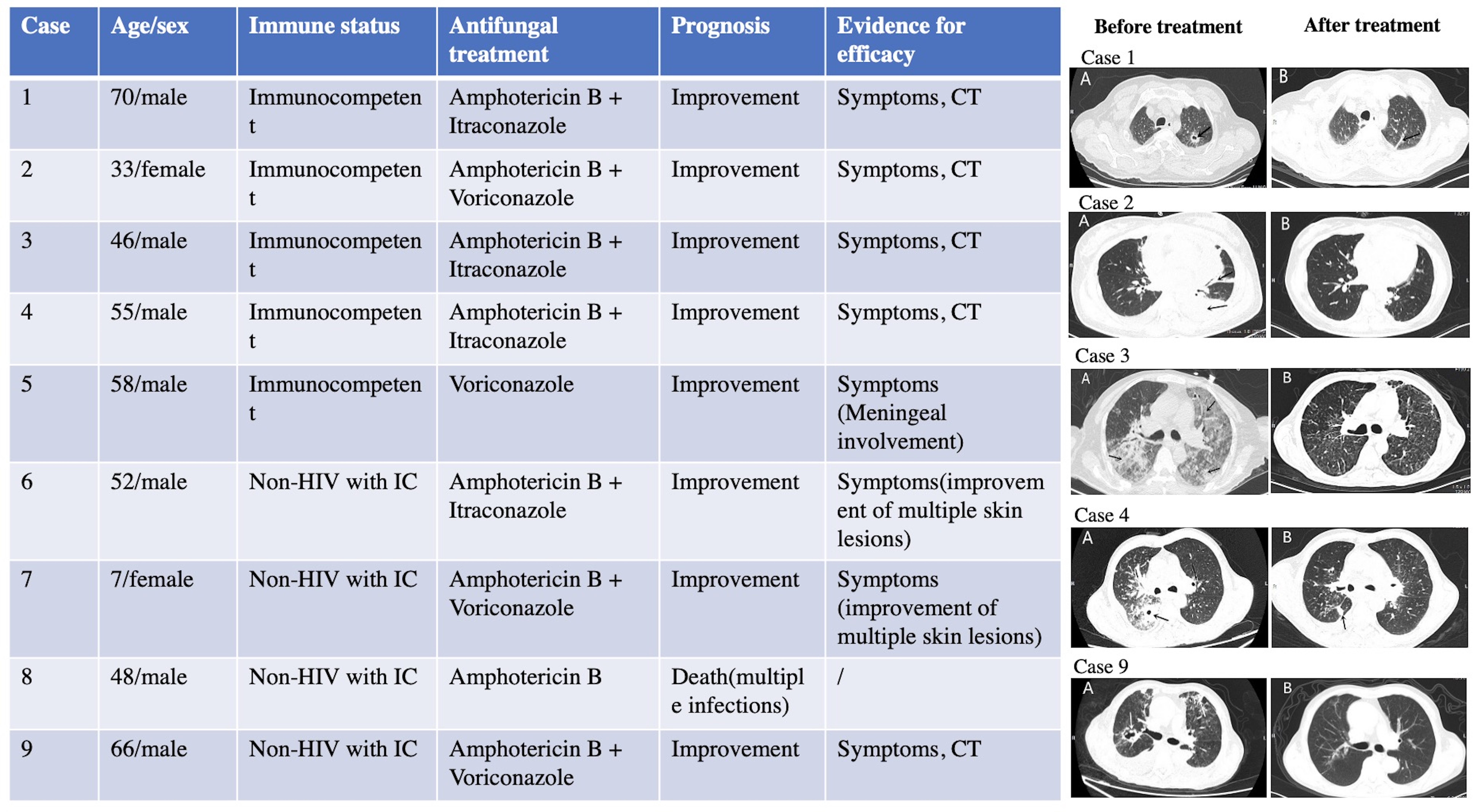

Supplement: Supplementary file 1 [file Image_1.JPEG]
